# Effects of *RHD* gene polymorphisms on distinguishing weak D or DEL from RhD− in blood donation in a Chinese population

**DOI:** 10.1002/mgg3.681

**Published:** 2019-04-05

**Authors:** Jie Shi, Ying Luo

**Affiliations:** ^1^ Nanjing Red Cross Blood Center Nanjing Jiangsu P. R. China; ^2^ Division of Nephrology and Rheumatology Center for Nephrology and Metabolomics Shanghai Tenth People's Hospital Tongji University School of Medicine Shanghai P. R. China

**Keywords:** polymorphism, *RHD*, RhD−

## Abstract

**Background:**

Weak D or DEL red blood cell units may be mistyped as RhD− by current serology assays, which can lead to incompatible transfusion to RhD− recipients and further cause anti‐D immunization. Molecular *RHD* blood group typing is a very effective method for overcoming current technical limits. The purpose of this study was to identify *RHD* single‐nucleotide polymorphisms (SNPs) and compare the genotype prevalence among confirmed RhD− individuals in a Chinese population as well as explore effective biomarkers for current weak D or DEL detection before blood transfusion.

**Methods:**

In the present study, 125 weak D (1, 2, 3, and 4.1) or DEL and 185 RhD− blood samples from donors detected by current standard serology were collected. Genotyping system was used to analyze the SNPs of *RHD* in each sample.

**Results:**

Seven SNPs (rs592372, rs11485789, rs6669352, rs3118454, rs1053359, rs590787, and rs3927482) were detected in the *RHD* region. Rs3118454, rs1053359, rs590787, and rs3927482 showed significant differences between the weak D (1, 2, 3 and 4.1) or DEL and RhD− groups. Further combined analysis of the allelic distribution of these four SNPs revealed their higher frequencies in the RhD− group.

**Conclusion:**

The SNPs rs3118454, rs1053359, rs590787, and rs3927482 in *RHD* showed a significantly higher frequency among an RhD− Chinese population and are potential biomarkers.

## 1 INTRODUCTION

Rh antigens are lipoprotein molecules sparsely located on the surface of erythrocytes (Xu, Zhang, Wang, Zhang, & Si, 2003). These antigens were first found in 1941 by Landsteiner and Weiner on the red cells of rhesus monkeys in India (Izetbegovic, 2013). During or after transplacental hemorrhage, Rh‐positive red blood cells in the fetus act as antigens in the mother's circulation. This pathogenesis of Rh alloimmunization which causes some hemolytic diseases in the fetus and newborn was confirmed by Chown in 1953 (Clausen, Damkjaer, & Dziegiel, 2014; Foudoulaki‐Paparizos et al., 2013; Gottvall & Filbey, 2008; Izetbegovic, 2013; Tiblad et al., 2013). Studies have shown that alloimmunity‐induced neonatal hemolysis is increased in RhD− multipara (Lubusky, Prochazka, Simetka, & Holuskova, 2013; Wang et al., 2014; Ye et al., 2014). The frequency of RhD− varies widely among ethnic groups, with the highest values observed in Europe at over 17% (Wagner, Kasulke, Kerowgan, & Flegel, 1995) but only 0.5% in Chinese (Peng et al., 2003) and Japanese (Okubo et al., 1991) populations. In Koreans, even lower frequencies of 0.15%–0.3% have been reported (Kim, Kim, Kim, Yon, & Park, 2005; Lee, 1965; Luettringhaus, Cho, Ryang, & Flegel, 2006). Although the prevalence of RhD− is much lower among Chinese people, the population of those with RhD− people was nearly 6.9 million in 2017. Therefore, accurate detection of the RhD blood type is very important.

Genetic factors have recently been recognized as important risk factors in the pathogenesis of many complex diseases such as hypertension, obesity, coronary artery disease, and diabetes (Joy, Lahiry, Pollex, & Hegele, 2008; Shore, 2008; Topol, Smith, Plow, & Wang, 2006; Wang, 2005). However, the genetic mechanisms of many diseases remain largely unknown. Single‐nucleotide polymorphisms (SNPs), as common DNA sequence variations, occur at single bases within genomic DNA and increase human genetic variation. SNPs are reported to be associated with many human diseases and in some cases are direct causative factors (Brookes, 1999; Hemminki & Bermejo, 2005; Yamada, 2008). Researchers have found that SNPs in candidate genes influenced individuals’ susceptibility to oncogenesis, including renal cell cancer (Cao et al., 2012; Purdue et al., 2011), acute lymphoblastic leukemia (Huang et al., 2012), esophageal cancer (Hildebrandt et al., 2009), bladder cancer(Chen et al., 2009), colon cancer, rectal cancer (Slattery et al., 2010), and lung cancer (Pu et al., 2011).These polymorphisms are located in specific coding or noncoding regions of genes and influence gene expression. However, whether SNPs exist in *RHD* (OMIM accession number: 111680) DNA sequences and whether they influence gene expression remains unclear.

In this study, we analyzed blood samples to identify potential SNPs in *RHD* using the HapMap system. We also conducted a SNaPshot assay to identify novel *RHD* SNPs and determined their association with RhD phenotypes in a Chinese population.

## 2 MATERIALS AND METHODS

### 2.1 Ethical compliance

The use of human blood samples in this study was approved by the independent ethics committee of Nanjing Red Cross Blood Center on 5 January 2011 (2011‐01). Blood samples used for SNP analysis were remaining samples after clinical use from blood donors who voluntarily donated whole blood at our blood center. All volunteers signed an informed consent statement to approve the use of their remaining sample.

### 2.2 Blood samples and immunohematology

Ethylenediaminetetraacetate‐anticoagulated blood samples were randomly collected from 300 blood donors at the Nanjing Red Cross Blood Center during a 3‐year period starting in January 2011. All donors were of Chinese Han ethnicity. Their Rh phenotypes were determined using standard serological kits according to the manufacturer's instructions (Gamma Biologicals, Houston, TX).

### 2.3 SNP selection

To assess the power for detecting associations due to linkage disequilibrium (LD) with causal loci, we carried out power calculations for an indirect association study that uses Tag‐SNPs. SNPs in *RHD* (GenBank reference sequence version number: NG_007494.1) were selected based on HapMap data (http://hapmap.ncbi.nlm.nih.gov/) and dbSNP data (http://www.ncbi.nlm.nih.gov/projects/SNP/). Potentially functional SNPs were identified according to the following criteria: (a) located in the 5′ flanking regions, 5′ untranslated region (UTR), 3′UTR, or coding regions with amino acid changes; or (b) minor allele frequency of 5% in the Chinese population. According to these criteria, seven SNPs were identified in *RHD*: rs11485789, rs1053359, rs3118454, rs590787, rs6669352, rs592372, and rs3927482.

### 2.4 DNA extraction and genotyping

Genomic DNA was extracted from the donated peripheral blood by proteinase K digestion and phenol‐chloroform extraction and stored at −80°C. Genotyping of these seven SNPs was performed using predesigned TaqMan SNP Genotyping Assays (Applied Biosystems, Foster City, CA). A 3.5‐μl reaction mixture containing 20 ng of genomic DNA, 10 μl of 2× TaqMan Genotyping Master Mix, 0.25 μl of the primers, probes mix, and 6.25 μl of double distilled water was prepared. Amplification was performed under the following conditions: 50°C for 2 min, 95°C for 10 min followed by 45 cycles at 95°C with 15 s for each cycle, and 60°C for 1 min. Amplification and analysis were performed in the 384‐well ABI 7900HT Real Time PCR System (Applied Biosystems) following the manufacturer's instructions. SDS 2.4 software (Applied Biosystems) was used for allelic discrimination. The genotyping rates of these SNPs were all above 98%. For quality control, four negative controls were included in each plate and 5% of the samples were randomly selected for repeated genotyping for confirmation; the results were 100% concordant (Cao et al., 2012).

### 2.5 Statistical analysis

Differences in the distributions of demographic characteristics, selected variables, and frequencies of genotypes between weak D or DEL and RhD− groups were tested using Student's *t*‐test (for continuous variables) or χ^2^‐test (for categorical variables). SNP frequencies in RhD− participants were tested against departure from Hardy–Weinberg equilibrium using a goodness‐of‐fit χ^2^‐test before further analysis. The associations between SNPs and weak D or DEL were estimated by computing the odds ratios (ORs) and 95% confidence intervals (CIs) from unconditional logistic regression analysis after adjusting for possible confounders. We used the Benjamini–Hochberg method to calculate the false discovery rate (FDR) and adjust the *P* value for multiple comparisons. The associations were considered statistically significant when the FDR‐adjusted *p* values were <0.05.

## 3 RESULTS

### 3.1 SNP statistics in *RHD*

**Figure 1:**
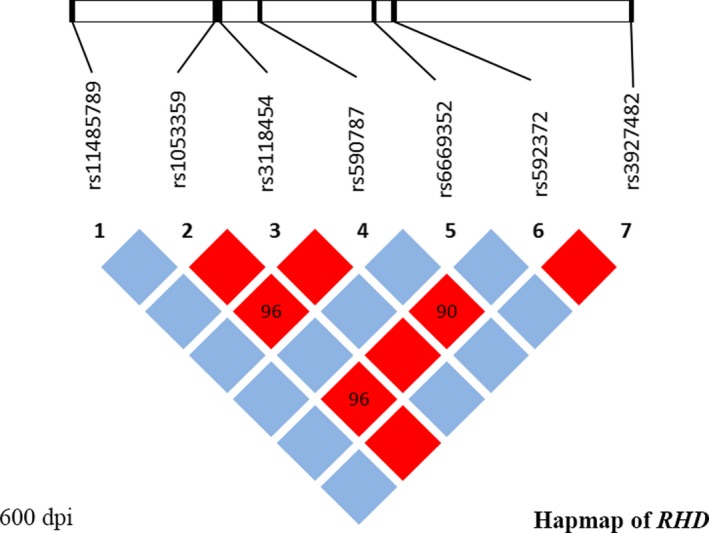
Hapmap of *RHD*. *RHD* GenBank reference sequence version number: NG_007494.1

The HapMap system was used to analyze the SNPs in *RHD* following the SNP selection principle described above. LD‐Plus was used to supplement the common Haploview style plot by providing additional statistical context for LD statistics. Multiple dimensions of genomic data could be displayed for easy comparison to evaluate the SNP relationships. As observed using Haploview software, seven SNPs in the *RHD* region (rs592372, rs11485789, rs6669352, rs3118454, rs1053359, rs590787, and rs3927482) were evaluated as the tag‐SNP of *RHD* (Figure 1).

### 3.2 Genotype and allele frequencies of SNPs in *RHD* in the weak D or DEL and RhD− groups

A genomic DNA assay based on a multiplex PCR coupled with a single base extension reaction (Silvy et al., 2011) was performed. This design allowed for simultaneous analysis of genotype and allele frequencies of the SNPs in *RHD* between the weak D or DEL and RhD− groups in the study populations. The general information of the donors and corresponding genotypes of *RHD* tag‐SNPs are listed in Table 1. To avoid the interference of confounding factors such as age and gender, we adjusted the data using a logistic regression model. The weak D or DEL and RhD− appeared to be adequately matched for age (*p *=* *0.173) and females (*p *=* *0.526). The genotype distributions and allele frequencies of the SNPs in *RHD* are presented in Table 1. For rs592372, rs11485789, and rs6669352, only one genotype of each SNP was detected in our donors, which could not be used to distinguish weak D or DEL from RhD−. Genotype frequencies in the weak D or DEL and RhD− donors for the remaining SNPs were in accordance with the Hardy–Weinberg equilibrium model, where *p *=* *0.569 for rs3118454, *p *=* *0.195 for rs1053359, *p *=* *0.413 for rs590787, and *p *=* *0.432 for rs3927482. As shown in Table 1, the genotype frequencies of rs3118454 were 8.8%, 22.4%, and 68.8% for the CC, CT, and TT genotypes among the weak D or DEL and 2.2%, 35.7%, and 62.1% among RhD−, respectively. The difference between the weak D or DEL and RhD− was significant (CT: *p *=* *0.001, CC: *p *=* *0.022). Additionally, the combined CT/TT genotype frequency was significantly lower in the weak D or DEL group than in the RhD− group (91.2% vs. 97.9%, *p *=* *0.008). When using the CC genotype as reference, we found that variant genotypes (CT and TT) were associated with a decreased trend compared to the TT genotype (*p*_trend_ = 0.003).

**Table 1:** Genotype and allele frequencies of the single‐nucleotide polymorphisms among the weak D or DEL and RhD−

Characters	Weak D or DEL *N* = 125 (*n*, %)	RhD− *N* = 185 (*n*, %)	*p*‐Value^a^	Adjusted OR (95% CI)^b^	Hardy‐Winberg equilibrium test
Age	32.24 ± 8.75	33.71 ± 9.59	0.173		
Sex					
Male	57	77			
Female	67	105	0.526		
rs592372					
GG	125	185	—	—	
rs11485789					
CC	125	185	—	—	
rs6669352					
GG	125	185	—	—	
rs3118454					0.569
CC	11 (8.8)	4 (2.2)		1.00	
CT	28 (22.4)	66 (35.7)	0.001	0.15 (0.04, 0.55)	
TT	86 (68.8)	115 (62.1)	0.022	0.32 (0.10, 1.04)	
CT/TT	114 (91.2)	181 (97.9)	0.008	0.26 (0.08, 0.85)	
*p*_trend_			0.003		
rs1053359					0.065
GG	31 (24.8)	105 (56.7)		1.00	
GC	57 (45.6)	69 (37.3)	<0.001	2.82 (1.62, 4.90)	
CC	37 (29.6)	11 (6.0)	<0.001	11.06 (5.03, 24.31)	
GC/CC	131	149	<0.001	4.04 (2.41, 6.76)	
*p*_trend_			<0.001		
rs590787					0.413
CC	92 (73.6)	64 (34.6)	<0.001	1.00	
CT	33 (26.4)	121 (65.4)		0.16 (0.10, 0.28)	
rs3927482					0.432
TT	117 (93.6)	145 (78.4)	<0.001	1.00	
TG	8 (6.4)	40 (21.6)		0.24 (0.11, 0.53)	

^a^Two‐sided χ^2^ test for distribution between cases and controls. ^b^Adjusted for age and sex in logistic regression model.

For rs1053359, the genotype frequencies were 56.8%, 37.3%, and 5.9% for the GG, GC, and CC genotypes among weak D or DEL and 24.8%, 45.6%, and 20.0% among RhD−, respectively. The difference between the weak D or DEL and RhD− groups was significant (GC: *p *=* *0.0001, CC: *p *<* *0.001). Additionally, the combined GC/CC genotype frequency was lower in the weak D or DEL group compared to the RhD− group (43.2% vs. 50.8%, *p *<* *0.0001). When using the TT genotype as a reference, we found that the variant genotypes (GC and CC) were associated with a lower frequency of CC compared to the TT genotype (adjusted OR = 2.82, 95% CI [1.62, 4.90] for GC, and OR = 11.06, 95% CI [5.03, 24.31] for CC; *p*_trend_ < 0.001). Similarly, we observed that the combined GC/CC genotypes were associated with a significantly lower frequency compared to the TT genotype (OR = 4.04, 95% CI [2.46, 6.76]).

The genotype frequencies of rs590787 were 73.6% for the CC genotypes among the weak D or DEL group and 34.6% among the RhD− group, respectively. The difference between the weak D or DEL and RhD− groups was significant (CC: *p *<* *0.001).

For the genotype of rs3927482, the frequencies for TT were 93.6% among the weak D or DEL group and 78.4% among the RhD− group. There was a significant difference between weak D or DEL and RhD− for rs3927482. Taken together, these data suggest that the *RHD* SNPs rs3118454, rs1053359, rs590787, and rs3927482 are putative biomarkers for weak D or DEL.

### 3.3 Frequency distributions of combined genotypes of SNPs between weak D or DEL and RhD− groups

Analysis of the allelic distribution revealed an association between rs3118454, rs1053359, rs590787, and rs3927482 with the phenotype of weak D or DEL (Table 1). To evaluate whether an interaction exists among these polymorphisms, we combined the four SNPs for analysis. As shown in Table 2, compared to subjects carrying 0 variant alleles, there were significant differences compared to subjects carrying 1 variant allele (*p *=* *0.004, adjusted OR = 0.33, 95% CI [0.15, 0.72]), 2–3 variant alleles (*p *<* *0.001, adjusted OR = 0.09, 95% CI [0.04, 0.21]), and 4–5 variant alleles (*p *<* *0.001, adjusted OR = 0.09, 95% CI [0.04, 0.20]). The *p* was <0.001, revealing a dose–response trend in the combined model of *RHD* SNPs.

**Table 2:** Frequency distributions of the combined model of tag‐single‐nucleotide polymorphisms between Weak D or DEL and RhD−

Numbers of risk alleles of the combined genotypes^a^	Weak D or DEL (*n* = 125)	RhD− (*n* = 185)	*p*‐value^c^	Adjusted OR (95% CI)^b^
0	37	11		1.00
1	49	45	0.004	0.33 (0.15, 0.72)
2–3	16	54	<0.001	0.09 (0.04, 0.21)
4–5	23	75	<0.001	0.09 (0.04, 0.20)
*p*_trend_			<0.001	

^a^Number represents the number of risk alleles within the combined genotypes; the risk alleles used for calculation were the rs3118454 T allele, rs1053359 G allele, rs590787 T allele and rs3927482 G allele. ^b^Adjusted for age and sex in logistic regression model. ^c^Two‐sided χ^2^ test for distribution between cases and controls.

## 4 DISCUSSION

In this study we investigated the potential role of the *RHD* in the phenotype of RhD. We identified seven SNPs in *RHD* and analyzed their frequencies in weak D or DEL and RhD− groups in a Chinese population.

Rh cDNA was first described by Avent in 1990 (Avent, Ridgwell, Tanner, & Anstee, 1990). Since then, many researchers have evaluated the antigens of the Rh system (Arce et al., 1993; Cherif‐Zahar et al., 1990; Le van Kim et al., 1992). In 1991, Colin established the first structure of the Rh locus (Colin et al., 1991). Numerous studies have confirmed that most RhD− individuals lack *RHD* (Hua et al., 2010; Pandey, Gautam, & Shukla, 1995; Wang et al., 2009; Xu et al., 2003). The mechanisms for the loss of *RHD* include gene conversion (Innan, 2003; Shao, Li, Xiong, Zhou, & Li, 2005), gene deletion (Avent et al., 1997; Chang et al., 1998; Fichou et al., 2012; Wagner & Flegel, 2000), antithetical missense mutations, and other missense mutations (Fichou et al., 2012). These mechanisms are associated with SNPs.

We observed a significant association between the SNPs in *RHD* and weak D or DEL individuals, specifically SNP rs3118454, rs1053359, rs590787, and rs3927482. However, the other three polymorphisms showed no effect on the phenotype of weak D or DEL subjects. We also analyzed the effects of the four *RHD* SNPs together. The results showed a significant association between the combined genotypes and RhD−. Subjects carrying 1–5 variant alleles were less prevalent than those carrying 0 variant alleles in RhD−. These results suggest that the selected four SNPs of *RHD* affect the phenotype of weak D or DEL individuals in the Chinese population.

In conclusion, we found that the SNPs rs3118454, rs1053359, rs590787, and rs3927482 in *RHD* were significantly associated with RhD phenotype in a Chinese population.

## CONFLICT OF INTEREST

The authors declare that there are no conflicts of interest in the present article.

## References

[mgg3681-bib-0001] Arce, M. A. , Thompson, E. S. , Wagner, S. , Coyne, K. E. , Ferdman, B. A. , & Lublin, D. M. (1993). Molecular cloning of RhD cDNA derived from a gene present in RhD‐positive, but not RhD‐negative individuals. Blood, 82, 651–655. 8329718

[mgg3681-bib-0002] Avent, N. D. , Liu, W. , Jones, J. W. , Scott, M. L. , Voak, D. , Pisacka, M. , … Fletcher, A. (1997). Molecular analysis of Rh transcripts and polypeptides from individuals expressing the DVI variant phenotype: An RHD gene deletion event does not generate All DVIccEe phenotypes. Blood, 89, 1779–1786. 9057663

[mgg3681-bib-0003] Avent, N. D. , Ridgwell, K. , Tanner, M. J. , & Anstee, D. J. (1990). cDNA cloning of a 30 kDa erythrocyte membrane protein associated with Rh (Rhesus)‐blood‐group‐antigen expression. Biochemical Journal, 271, 821–825. 10.1042/bj2710821 2123099 PMC1149638

[mgg3681-bib-0004] Brookes, A. J. (1999). The essence of SNPs. Gene, 234, 177–186. 10.1016/S0378-1119(99)00219-X 10395891

[mgg3681-bib-0005] Cao, Q. , Ju, X. , Li, P. , Meng, X. , Shao, P. , Cai, H. , … Yin, C. (2012). A functional variant in the MTOR promoter modulates its expression and is associated with renal cell cancer risk. PLoS ONE, 7, e50302 10.1371/journal.pone.0050302 23209702 PMC3508984

[mgg3681-bib-0006] Chang, J. G. , Wang, J. C. , Yang, T. Y. , Tsan, K. W. , Shih, M. C. , Peng, C. T. , & Tsai, C. H. (1998). Human RhDel is caused by a deletion of 1,013 bp between introns 8 and 9 including exon 9 of RHD gene. Blood, 92, 2602–2604. 9746809

[mgg3681-bib-0007] Chen, M. , Cassidy, A. , Gu, J. , Delclos, G. L. , Zhen, F. , Yang, H. , … Wu, X. (2009). Genetic variations in PI3K‐AKT‐mTOR pathway and bladder cancer risk. Carcinogenesis, 30, 2047–2052. 10.1093/carcin/bgp258 19875696 PMC2792319

[mgg3681-bib-0008] Cherif‐Zahar, B. , Bloy, C. , Le Van Kim, C. , Blanchard, D. , Bailly, P. , Hermand, P. , … Colin, Y. (1990). Molecular cloning and protein structure of a human blood group Rh polypeptide. Proceedings of the National Academy of Sciences of the United States of America, 87, 6243–6247. 10.1073/pnas.87.16.6243 1696722 PMC54509

[mgg3681-bib-0009] Clausen, F. B. , Damkjaer, M. B. , & Dziegiel, M. H. (2014). Noninvasive fetal RhD genotyping. Transfusion and Apheresis Science, 50, 154–162. 10.1016/j.transci.2014.02.008 24642067

[mgg3681-bib-0010] Colin, Y. , Cherif‐Zahar, B. , Le Van Kim, C. , Raynal, V. , Van Huffel, V. , & Cartron, J. P. (1991). Genetic basis of the RhD‐positive and RhD‐negative blood group polymorphism as determined by Southern analysis. Blood, 78, 2747–2752. 1824267

[mgg3681-bib-0011] Fichou, Y. , Chen, J. M. , Le Marechal, C. , Jamet, D. , Dupont, I. , Chuteau, C. , … Ferec, C. (2012). Weak D caused by a founder deletion in the RHD gene. Transfusion, 52, 2348–2355. 10.1111/j.1537-2995.2012.03606.x 22420867

[mgg3681-bib-0012] Foudoulaki‐Paparizos, L. , Valsami, S. , Bournas, N. , Tsantes, A. , Grapsas, P. , Mantzios, G. , … Politou, M. (2013). Alloimmunisation during pregnancy in Greece: Need for nationwide HDFN prevention programme. Transfusion Medicine (Oxford, England), 23, 254–259. 10.1111/tme.12063 23826966

[mgg3681-bib-0013] Gottvall, T. , & Filbey, D. (2008). Alloimmunization in pregnancy during the years 1992–2005 in the central west region of Sweden. Acta Obstetricia et Gynecologica Scandinavica, 87, 843–848. 10.1080/00016340802268880 18704776

[mgg3681-bib-0014] Hemminki, K. , & Bermejo, J. L. (2005). Relationships between familial risks of cancer and the effects of heritable genes and their SNP variants. Mutation Research, 592(1–2), 6–17. 10.1016/j.mrfmmm.2005.05.008 15990124

[mgg3681-bib-0015] Hildebrandt, M. A. , Yang, H. , Hung, M. C. , Izzo, J. G. , Huang, M. , Lin, J. , … Wu, X. (2009). Genetic variations in the PI3K/PTEN/AKT/mTOR pathway are associated with clinical outcomes in esophageal cancer patients treated with chemoradiotherapy. Journal of Clinical Oncology, 27, 857–871. 10.1200/JCO.2008.17.6297 19164214 PMC2738430

[mgg3681-bib-0016] Hua, X. , Shi‐Hui, Y. , Chao‐Peng, S. , He‐Xiang, X. , Jian‐Geng, Z. , Nai‐Bao, Z. , & Chen, H. (2010). A new RHD‐positive, D antigen negative allele in Chinese. Vox Sanguinis, 98, 576 10.1111/j.1423-0410.2009.01280.x 20136792

[mgg3681-bib-0017] Huang, L. , Huang, J. , Wu, P. , Li, Q. , Rong, L. , Xue, Y. , … Fang, Y. (2012). Association of genetic variations in mTOR with risk of childhood acute lymphoblastic leukemia in a Chinese population. Leukaemia & Lymphoma, 53, 947–951. 10.3109/10428194.2011.628062 21973240

[mgg3681-bib-0018] Innan, H. (2003). A two‐locus gene conversion model with selection and its application to the human RHCE and RHD genes. Proceedings of the National Academy of Sciences of the United States of America, 100, 8793–8798. 10.1073/pnas.1031592100 12857961 PMC166392

[mgg3681-bib-0019] Izetbegovic, S. (2013). Occurrence of ABO And RhD incompatibility with Rh negative mothers. Mater Sociomed, 25, 255–258. 10.5455/msm.2013.25.255-258 24511269 PMC3914752

[mgg3681-bib-0020] Joy, T. , Lahiry, P. , Pollex, R. L. , & Hegele, R. A. (2008). Genetics of metabolic syndrome. Current Diabetes Reports, 8, 141–148. 10.1007/s11892-008-0025-y 18445357

[mgg3681-bib-0021] Kim, J. Y. , Kim, S. Y. , Kim, C. A. , Yon, G. S. , & Park, S. S. (2005). Molecular characterization of D‐ Korean persons: Development of a diagnostic strategy. Transfusion, 45, 345–352. 10.1111/j.1537-2995.2005.04311.x 15752151

[mgg3681-bib-0022] Le van Kim, C. , Mouro, I. , Cherif‐Zahar, B. , Raynal, V. , Cherrier, C. , Cartron, J. P. , & Colin, Y. (1992). Molecular cloning and primary structure of the human blood group RhD polypeptide. Proceedings of the National Academy of Sciences of the United States of America, 89, 10925–10929. 10.1073/pnas.89.22.10925 1438298 PMC50455

[mgg3681-bib-0023] Lee, S. Y. (1965). Further analysis of Korean blood types. Yonsei Medical Journal, 6, 16–25. 10.3349/ymj.1965.6.1.16 5870360

[mgg3681-bib-0024] Lubusky, M. , Prochazka, M. , Simetka, O. , & Holuskova, I. (2013). Guideline for prevention of RhD alloimmunizationin RhD negative women. Ceska Gynekologie, 78, 132–133. 23710977

[mgg3681-bib-0025] Luettringhaus, T. A. , Cho, D. , Ryang, D. W. , & Flegel, W. A. (2006). An easy RHD genotyping strategy for D‐ East Asian persons applied to Korean blood donors. Transfusion, 46, 2128–2137. 10.1111/j.1537-2995.2006.01042.x 17176325

[mgg3681-bib-0026] Okubo, Y. , Seno, T. , Yamano, H. , Yamaguchi, H. , Lomas, C. , & Tippett, P. (1991). Partial D antigens disclosed by a monoclonal anti‐D in Japanese blood donors. Transfusion, 31, 782 10.1046/j.1537-2995.1991.31892023512.x 1926326

[mgg3681-bib-0027] Pandey, M. , Gautam, A. , & Shukla, V. K. (1995). ABO and Rh blood groups in patients with cholelithiasis and carcinoma of the gall bladder. British Medical Journal, 310, 1639 10.1136/bmj.310.6995.1639 PMC2550011 7795450

[mgg3681-bib-0028] Peng, C. T. , Shih, M. C. , Liu, T. C. , Lin, I. L. , Jaung, S. J. , & Chang, J. G. (2003). Molecular basis for the RhD negative phenotype in Chinese. International Journal of Molecular Medicine, 11, 515–521. 12632107

[mgg3681-bib-0029] Pu, X. , Hildebrandt, M. A. , Lu, C. , Lin, J. , Stewart, D. J. , Ye, Y. , … Wu, X. (2011). PI3K/PTEN/AKT/mTOR pathway genetic variation predicts toxicity and distant progression in lung cancer patients receiving platinum‐based chemotherapy. Lung Cancer, 71(1), 82–88. 10.1016/j.lungcan.2010.04.008 20447721 PMC2952281

[mgg3681-bib-0030] Purdue, M. P. , Johansson, M. , Zelenika, D. , Toro, J. R. , Scelo, G. , Moore, L. E. , … Brennan, P. (2011). Genome‐wide association study of renal cell carcinoma identifies two susceptibility loci on 2p21 and 11q13.3. Nature Genetics, 43, 60–65. 10.1038/ng.723 21131975 PMC3049257

[mgg3681-bib-0031] Shao, C. P. , Li, Z. , Xiong, W. , Zhou, Y. Y. , & Li, X. M. (2005). [Generation of RHD‐CE(2‐9)‐D allele by gene conversion in cis]. Yi Chuan, 27, 561–565. 16120578

[mgg3681-bib-0032] Shore, S. A. (2008). Obesity and asthma: Possible mechanisms. Journal of Allergy and Clinical Immunology, 121, 1087–1093; quiz 1094–1085. 10.1016/j.jaci.2008.03.004 18405959

[mgg3681-bib-0033] Silvy, M. , Simon, S. , Gouvitsos, J. , Di Cristofaro, J. , Ferrera, V. , Chiaroni, J. , & Bailly, P. (2011). Weak D and DEL alleles detected by routine SNaPshot genotyping: Identification of four novel RHD alleles. Transfusion, 51, 401–411. 10.1111/j.1537-2995.2010.02830.x 20723165

[mgg3681-bib-0034] Slattery, M. L. , Herrick, J. S. , Lundgreen, A. , Fitzpatrick, F. A. , Curtin, K. , & Wolff, R. K. (2010). Genetic variation in a metabolic signaling pathway and colon and rectal cancer risk: mTOR, PTEN, STK11, RPKAA1, PRKAG2, TSC1, TSC2, PI3K and Akt1. Carcinogenesis, 31, 1604–1611. 10.1093/carcin/bgq142 20622004 PMC2930805

[mgg3681-bib-0035] Tiblad, E. , Taune Wikman, A. , Ajne, G. , Blanck, A. , Jansson, Y. , Karlsson, A. , … Westgren, M. (2013). Targeted routine antenatal anti‐D prophylaxis in the prevention of RhD immunisation–outcome of a new antenatal screening and prevention program. PLoS ONE, 8, e70984 10.1371/journal.pone.0070984 23940682 PMC3735499

[mgg3681-bib-0036] Topol, E. J. , Smith, J. , Plow, E. F. , & Wang, Q. K. (2006). Genetic susceptibility to myocardial infarction and coronary artery disease. Human Molecular Genetics, 15, R117–R123. 10.1093/hmg/ddl183 16987874

[mgg3681-bib-0037] Wagner, F. F. , & Flegel, W. A. (2000). RHD gene deletion occurred in the Rhesus box. Blood, 95, 3662–3668. 10845894

[mgg3681-bib-0038] Wagner, F. F. , Kasulke, D. , Kerowgan, M. , & Flegel, W. A. (1995). Frequencies of the blood groups ABO, Rhesus, D category VI, Kell, and of clinically relevant high‐frequency antigens in south‐western Germany. Infusionstherapie und Transfusionsmedizin, 22, 285–290. 8924742 10.1159/000223144

[mgg3681-bib-0039] Wang, Q. (2005). Molecular genetics of coronary artery disease. Current Opinion in Cardiology, 20, 182–188. 10.1097/01.hco.0000160373.77190.f1 15861005 PMC1579824

[mgg3681-bib-0040] Wang, Q. P. , Dong, G. T. , Wang, X. D. , Gu, J. , Li, Z. , Sun, A. Y. , … Shan, P. N. (2014). An investigation of secondary anti‐D immunisation among phenotypically RhD‐negative individuals in the Chinese population. Blood Transfusion, 12, 238 10.2450/2013.0184-12 23399369 PMC4039707

[mgg3681-bib-0041] Wang, X. D. , Wang, B. L. , Ye, S. L. , Liao, Y. Q. , Wang, L. F. , & He, Z. M. (2009). Non‐invasive foetal RHD genotyping via real‐time PCR of foetal DNA from Chinese RhD‐negative maternal plasma. European Journal of Clinical Investigation, 39, 607–617. 10.1111/j.1365-2362.2009.02148.x 19545247

[mgg3681-bib-0042] Xu, Q. , Zhang, J. , Wang, Q. , Zhang, S. , & Si, G. (2003). RHD gene polymorphism among RhD‐negative Han Chinese. Chinese Medical Journal, 116, 1539–1543. 14570619

[mgg3681-bib-0043] Yamada, R. (2008). Primer: SNP‐associated studies and what they can teach us. Nature Clinical Practice Rheumatology, 4, 210–217. 10.1038/ncprheum0757 18319711

[mgg3681-bib-0044] Ye, S. H. , Wu, D. Z. , Wang, M. N. , Wu, X. Y. , Xu, H. G. , Xu, H. , & Shao, C. P. (2014). A comprehensive investigation of RHD polymorphisms in the Chinese Han population in Xi'an. Blood Transfusion, 12, 396 10.2450/2013.0121-13 24333088 PMC4111822

